# Pediatric Hepatic Angiosarcoma (PHAS) and Vinyl Chloride—A Ghost of the Past May Loom over East Palestine, OH, and beyond: A Critical Commentary

**DOI:** 10.3390/diagnostics13081412

**Published:** 2023-04-14

**Authors:** Consolato M. Sergi

**Affiliations:** 1 Anatomic Pathology Division, Children’s Hospital of Eastern Ontario (CHEO), University of Ottawa, 401 Smyth Road, Ottawa, ON K1H 8L1, Canada; csergi@cheo.on.ca; Tel.: +1-613-737-7600; Fax: +1-613-738-4837; 2 Department of Laboratory Medicine and Pathology, University of Alberta, 8440 112 St., Edmonton, AB T6G 2B7, Canada

**Keywords:** vinyl chloride, liver, angiosarcoma, tumor, environment

## Abstract

Road accidents are not infrequent everywhere in the world, but when they involve poisonous and dangerous chemical compounds, they represent a hazard and an issue for public health. In this commentary, we briefly review a recent East Palestine event and one of the chemicals primarily involved with a predisposition to initiate a carcinogenetic process. The author reviewed, as a consultant, numerous chemical compounds for the International Agency for Research on Cancer, a trusted agency of the World Health Organization. Something is looming over the territories of East Palestine, Ohio, United States, draining water from the soil. We speculate that there is a dark and opprobrious fate for this area of the United States due to the potential increase in cases of pediatric hepatic angiosarcoma, which will also be revised in this commentary.

## 1. Introduction

In the last century, train derailments have been much more common and aggravated by fatalities and environmental impacts. The causes of a train derailment may be complex and complicated. They include a derailment, i.e., a train running off its rail. This occurs due to a collision with an oncoming object, an error of conduction by the locomotive, a failure of a mechanical track origin, damaged rails, or a partial or full defect of the wheels. A derailment does not unavoidably mean that the train departs from the tracks because the damage could be minimal and accentuated by changes in the climate conditions. On the other hand, a severe derailment can shatter if it happens while the train is inescapably moving at a high speed. In North America, over four-fifths of railroad crossings have no adequate warning devices (e.g., lights and gates), and more than 50% of all railroad accidents occur at unprotected railroad crossings. Car accidents at unguarded railroad crossings include several conditions. They often include (single) driver distraction, driver(s) inebriation/intoxication, poor visibility, excessive or inadequate speed for the railroad, electronic signal malfunctioning, obstacles that obstruct a driver’s or both drivers’ view, and an inability or delay in sounding an alarm by the driver when facing a removable block. A recent accident involving a train derailment at East Palestine, Ohio, United States, has risen to worldwide concern about safety and quality controls in this industry and brings back memories of dioxin disasters in Missouri, India, and Italy [1]. Although all of the causes are still to be evaluated thoroughly, there has been an enormous environmental impact that may have intergenerational consequences. The residents of East Palestine have been told that it is safe to return home. However, questions remain about prolonged exposure in the air, in the water, and probably more in the soil, even at low levels. In fact, the train derailment was associated with the disastrous environmental impact of vinyl chloride, butyl acrylate, isobutylene, ethylene glycol, and ethylhexyl acrylate.

Butyl acrylate is a colorless compound in a liquid state. It harbors a robust and fruity odor. Butyl acrylate is frequently used in the industry to produce various coatings, plastics, polymers, and resins. According to the United States-based Center for Disease Control (CDC), exposure to the chemical can cause irritation to the eyes and skin, rashes, and breathing difficulties. Isobutylene is a colorless compound in a gaseous state. It is often used to produce packaging materials, plastics, and resins. Its exposure can provoke symptoms in humans, such as dizziness and headaches. Ethylene glycol is a synthetic chemical compound used in antifreeze, hydraulic brake fluids, inks, and painting mixtures. Ethylhexyl acrylate is a colorless liquid compound often used to produce several plastics and different polymers. Both ethylene glycol and ethylhexyl acrylate can irritate the eyes, skin, and respiratory tract, with individuals experiencing nausea and vomiting at high concentrations. Overall, vinyl chloride remains the primary compound of concern. Vinyl chloride is a colorless compound and a flammable gas [2]. Vinyl chloride monomer (VCM) is issued to produce polyvinyl chloride (PVC) plastic for packaging materials and vinyl products used in the electronic, medical, and building industries. VCM exposure is characterized by drowsiness, disorientation, numbness, and tingling of the limbs. Moreover, VCM exposure induces nausea and vomiting, but this compound has the ominous fate of triggering liver cancer, mainly hepatic angiosarcoma, in both experimental animals and humans.

## 2. Vinyl Chloride

Vinyl chloride, or vinyl chloride monomer (VCM), is a colorless, gaseous compound at room temperature [3,4]. As indicated above, PVC is the polymerized form of VCM. PVC is widely used in the plastics industry. VCM is a manufactured compound found nearly exclusively with manufacturers making PVC. There is solid evidence that small amounts of VCM are identified in finished plastic products. The highest concentration has been found in vinyl musical records [5]. However, VCM is also ubiquitous because it is present in cigarette smoke, which can affect both active and passive smokers. The amount of VCM depends exclusively on the chloride concentration of the tobacco. Vinyl chloride has been produced in the United States of America (USA) for more than 90 years [6,7,8]. It has been determined that about 80,000 workers in the USA and 40,000 workers in Europe have been possibly exposed to VCM up to 1997. VCM is etiologically associated with the progress of non-cirrhotic portal hypertension, which is related to sinusoidal endothelial damage. Additionally, it has been associated with a particular malignant mesenchymal tumor of the liver, or hepatic angiosarcoma, other than hepatocellular carcinoma (HCC) [7,9,10].

The International Agency for Research on Cancer (IARC) has concluded that there is sufficient evidence that VCM is the etiology of HCC. In 1978, in Europe, the continental producers of VCM recognized the need to create a registry of angiosarcoma cases. This registry has been vital in disclosing a decreasing annual number of cases since its inception. There are no new cases in which the exposure started after 1972. In 2009, the register included 231 patients. Most of the affected individuals arise from Europe and North America, but some also come from Eastern Europe or China. However, the inadequacy of reporting, rather than the stringent safety precautions, has been advocated for some parts of the world. In the USA, the annual incidence of hepatic angiosarcoma was 0.014 per 100,000 workers [7,11,12,13,14,15,16]. The mean latency between exposure and the development of hepatic angiosarcoma in the registry was 27 years. Before environmental controls, the exposure limits were considered 7.8 g/m^3^ (10 ppm equals about 26 mg/m^3^). Still, recently, in countries with strict and strictly enforced environmental standards, the exposure levels are often less than 1 mg/m^3^. In countries where the environmental controls are less strictly regulated, high levels of exposure (e.g., up to 0.8 g/m^3^) still occur. VCM is quickly absorbed through the respiratory tract and is metabolized rapidly by the liver. Chloroethylene oxide is a reactive metabolite that occurs in the intermediate steps. It is detoxified by conjugation with glutathione or aldehyde dehydrogenase. On the other hand, chloroethylene oxide can build DNA adducts that are highly mutagenic. There is compelling literature, and VCM has been demonstrated to be genotoxic in vivo in studies in rats [17]. VCM causes DNA strand breaks, micronucleus formation, sister chromatid exchanges, and other chromosomal aberrations and has been found mutagenic in numerous in vitro assays. In hepatic angiosarcoma, mutations of the Ki-ras-2 and p53 genes have been identified following VCM exposure, despite the fact that some of these mutations do not seem to be VCM characteristics. The mutations include changes in the Ki-ras-2 gene at codon 13 (G -> A) or at codon 12 (G -> A), in addition to mutations found at several different positions on the p53 gene, with about half of the vinyl chloride-associated neoplasms harboring the A:T to T:A transversion in the *TP53* gene [16].

Maltoni and Cotti did not identify a dose–response relationship between VCM and the oncogenic development of HCC [18]. HCC only occurred at a dose that was several times the amount of exposure in humans. In 1983, Drew et al. [19] pointed out an increased rate of liver cell adenomas and HCC in VCM-exposed rodents (rats). However, there was no apparent dose–response relationship. Feron et al. [20] and Til et al. [21] also identified dose–response relationships between the degree of exposure to VCM and the oncogenic development of HCC. Both cancer bioassays and humans show similar features [7,8,10]. The injury is located in the sinusoids, despite the fact that hepatocytes may also indicate an oncogenetic transformation. In mild cases, there may be exclusively sinusoidal dilatation, which could be associated with hyperplasia of the endothelial cells. Perisinusoidal and perivascular fibrosis are also noted. Later, periportal, portal, and subcapsular fibrosis are seen. If the fibrosis is massive and coalescent, cirrhosis is diagnosed. Regarding neoplasia, endothelial cells disclose an enlargement of the nuclei, which can harbor membrane irregularities and hyperchromatism. These findings, in the absence of an invasion, portend the diagnosis of dysplasia. In the presence of aggressivity and cell proliferation, the diagnosis of neoplasm is made. The evidence for VCM in the etiology of cirrhosis, HCC, and hepatic angiosarcoma is solid and corroborated by the IARC/WHO evaluation, despite the fact that all of these studies are open to disapproval on different grounds. These include bias, as in the Taiwan study. Moreover, in the 1960s and 1970s, the diagnosis of cirrhosis was often made exclusively clinically without any biopsy data. It is also valuable considering the dose–response relationship between VCM and HCC. If the dose–response relationship is real, this is indeed support for a causal relationship association, but an European study evaluated the dose–response relationship in only ten patients affected with HCC. Finally, patients included in the multicenter studies were mainly diagnosed before testing for infections with hepatotropic viruses was available, and alcohol consumption was poorly recorded. Thus, although a trustworthy and leading organization (the IARC) has indicated that VCM does cause neoplasms, the basis for these statements may need further investigation in the future.

## 3. Pediatric Hepatic Angiosarcoma

**Figure 1:**
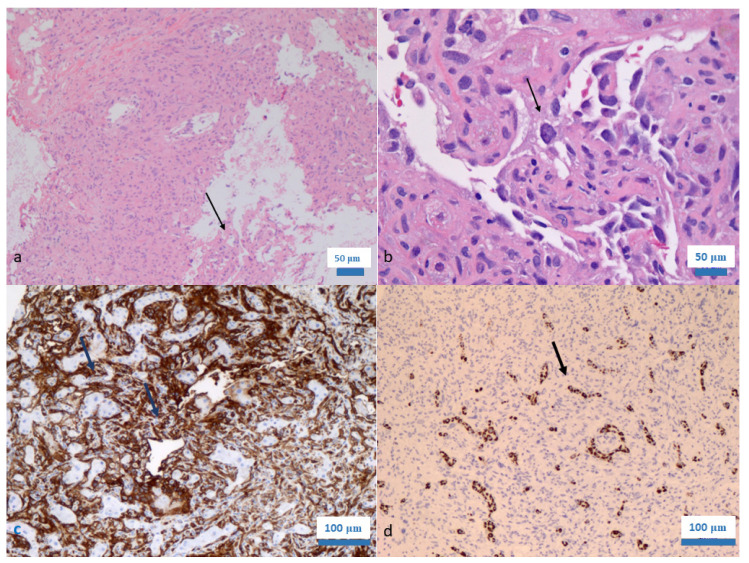
(**a**) Hepatic angiosarcoma of the youth with aggressive infiltration (arrow) of the surrounding liver parenchyma (hematoxylin and eosin staining; ×100); (**b**) The high power of the hepatic angiosarcoma demonstrated in (**a**) showing vascular channels decorated by atypical mesenchymal cells (arrow) (hematoxylin and eosin staining; ×400); (**c**) Positive expression of CD31 in the tumor cells (anti-CD31 avidin–biotin complex immunohistochemistry; ×200); (**d**) Negative expression of HepPar1 in the tumor cells showing focal expression in the liver cells only (HepPar1 avidin–biotin complex immunohistochemistry; ×200).

Pediatric hepatic angiosarcoma (PHAS) is a rare neoplasm, with Potanos et al. reporting less than 50 cases described in the literature [22]. PHAS happens twice as often in children or youth of the female sex. The stated age at clinical diagnosis varies from eight weeks to fifteen years [11,12,23,24,25]. Although ecological exposures have been associated with hepatic angiosarcoma in adults of all ages, only a single case of PHAS has been supposedly linked to arsenic (As) exposure [7,26,27]. In fact, these chemicals necessitate several years of toxic exposure prior to tumor detection. The signs and symptoms of PHAS vary from jaundice, abdominal pain, and vomiting to fever, tachypnea, and dyspnea. However, it may mirror the age presentation as well. Skeletal, pulmonary, adrenal, renal, and lymphatic metastases can be encountered. During histopathological investigations, PHAS is indistinguishable from its adult counterpart (Figure 1).

However, studies have reported some features of the hypercellular fiber bundles and whorls of atypical spindle-shaped endothelial cells harboring the cytoplasmic eosinophilic globules, which are more often encountered in PHAS than in adult hepatic angiosarcoma. PHAS can have histological fields of miniature channels with bland or minimally atypical endothelium. It may mimic infantile hepatic hemangiomas (solitary, diffuse, or multifocal types, according to the United States-based Hepatic Tumor Registry Classification) or reactive vasculature around another tumor; thus, limited sampling of a lesion may be inconclusive or misleading. Some pediatric pathologists diagnose atypical hepatic vascular tumors in their reports because they do not stretch to the level of an evident angiosarcoma, such as type 2 infantile hemangioendothelioma, as described previously. Different from the benign-appearing infantile hemangioendothelioma type 1 category, category type 2 exhibits more aggressive features, showing branching vascular structures, irregular tumor budding, and tortuous endothelial vascular spaces lined by larger and more hyperchromatic and pleomorphic cells. The average recognized survival time for PHAS is less than 16 months. The use of inhibitors to target the vascular endothelial growth factor (VEGF) has been investigated in subjects harboring sarcomas and was especially convincing in treating angiosarcomas, given the high rate of VEGF expression in these neoplasms [28,29,30,31,32]. In adult patients, a Phase III investigation of paclitaxel/bevacizumab in breast cancer with metastatic disease and a Phase II investigation likening bevacizumab plus carboplatin and paclitaxel with carboplatin and paclitaxel alone established the potential advantage of this combination in refractory solid tumors. In a nutshell, the differential diagnosis of liver angiosarcomas include inflammatory diseases of the liver (no specific neoplasm; inflammatory markers are critical), benign vascular disorders of the liver (no atypia, no invasion, and no necrosis), Kaposi sarcoma (HIV positivity, portal distribution, no angiosarcomatous foci, and presence of extrahepatic sarcoma nodules), fibrosarcoma (prominent fibrocellular stroma and CD31 negative), hepatic metastasis from the angiosarcoma arising in other organs (staging-aimed diagnostic imaging positive for extrahepatic vascular neoplasm), epithelioid hemangioendothelioma (minimal or focal atypia, no or minimal mitotic activity, no or minimal necrosis, and often marked with fibrous and hyalinized stroma), cholangiocellular carcinoma (the epithelial markers of bile duct differentiation are often positive), and of course, hepatocellular carcinoma (atypical hepatocytes, unremarkable endothelial cells, and HepPar1 positivity).

## 4. Public Health Considerations

Sadly, therapeutic guidelines have not been definitively established due to the small number of cases of this rare malignancy. Diagnosing liver angiosarcomas remains challenging due to the non-specificity of the stated symptoms, such as body weight loss and abdominal discomfort. Laboratory markers for neoplasms are likewise non-specific to the diagnosis, and identifying the malignant tumor using radiological imaging is challenging. A biopsy of the lesion carries the risk of intralesional or intraabdominal bleeding due to the high vascularity of the neoplasm. In order to establish such a challenging diagnosis, a cooperative effort must be assembled between primary care doctors, surgeons, hepatologists, radiologists, laboratory physicians, and pathologists. Once the diagnosis of hepatic angiosarcomas is certain, the prognosis continues to be very poor, and the therapeutic approach would be surgery with an attempt to achieve R0 (no residual disease) versus palliative care. No unique therapeutic guidelines have been established to date due to the rarity of the cancer. An algorithm for a therapeutic strategy for liver angiosarcomas remains challenging, even in the 21st century. Surgery with no residual malignant disease is the cornerstone, but there is a substantial difficulty in achieving the well-aimed and hoped-for R0. Thus, even after an extensive surgical approach, the rate of recurrence is high, ranging from 30% to 100%. To control this despicable situation, adjuvant radiotherapy is used in patients with negative microscopic margins (R0) or even in unresectable cases. Cytotoxic chemotherapy is the predominant method of treatment for angiosarcomas exhibiting metastases [33]. It includes doxorubicin (and liposomal doxorubicin), ifosfamide, and taxanes. Doxorubicin remains the main anthracycline drug for soft-tissue sarcomas. It provides a median overall survival of 8–14 months and a decreased rate of metastasis. The combination of doxorubicin and ifosfamide has shown improved survival rates for malignant mesenchymal tumors compared to single-agent anthracyclines. In addition, olaratumab seems to harbor both better progression-free and overall survival in combination with doxorubicin for the first-line treatment of angiosarcomas. Olaratumab is a recombinant human immunoglobulin G subclass 1 (IgG1) monoclonal antibody. Finally, paclitaxel is considered an active monotherapy for angiosarcomas and is often used in the first or second line for metastatic disease. However, there is still controversy regarding the optimal selection and sequence of anthracycline and taxane-based chemotherapy. Recently, proangiogenic growth factors and their receptors, including the vascular endothelial growth factor (VEGF) and platelet-derived growth factor (PDGF), have been demonstrated as useful targets in cancer progression. The inhibition of the VEGF/VEGFR signaling pathway is performed by tyrosine kinase inhibitors (TKIs), particularly sorafenib and pazopanib. Moreover, non-selective beta blockers, such as propranolol, seem to be useful at least in some cases, with an increase in the survival rate. Additionally, programmed death 1 (PD-1) and its receptors, including ligand-1 (PD-L1) and ligand-2 (PD-L2), have been widely used in therapeutic strategies for malignant tumors. The curative effect of the anti-PD-1 antibody for angiosarcomas was identified in small trials [33].

**Figure 2:**
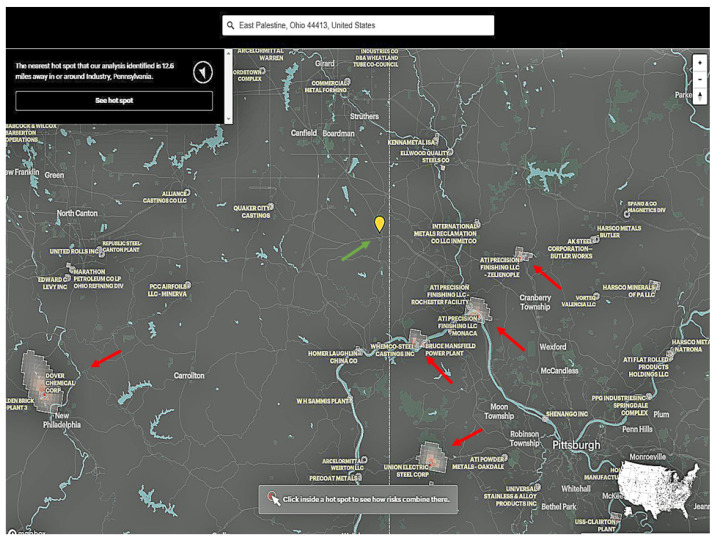
ToxMap showing the area of East Palestine, Ohio (green arrow) and five industrial establishments in the neighborhood, four of which are quite close to Pittsburgh, PA, United States of America. Epidemiologists and public health officials may want to keep this current ToxMap and subsequent data in mind by analyzing the rate of liver tumors in Ohio and Pennsylvania in the near future. ToxMap is a web-based geographic information system (GIS) (http://toxnet.nlm.nih.gov, accessed on 25 March 2023). The red arrows indicate industrial companies that may have an environmental impact in the areas surrounding East Palestine.

Chemical pollution and other pesticides may be responsible for childhood tumors [34,35,36,37,38,39,40,41,42,43]. In an analysis of the toxicologic exposure in East Palestine, there was no apparent chemical burden for the population living in that district (Figure 2). Epidemiologists and public health officials may want to keep this current ToxMap and subsequent data in mind by analyzing the rate of liver tumors in Ohio and Pennsylvania in the near future. ToxMap is a web-based geographic information system (GIS) that provides a GIS-based toxicologic map arising from an extensive collection of environmental health information, including both technical and bibliographical data on hazardous chemical compounds (http://toxnet.nlm.nih.gov, accessed on 25 March 2023). This commentary is a warning to healthcare officials, who may need to follow up on this population for a few decades.

Overall, despite the fact that around 75% of tumors have no known etiology, the most common etiologic factors of angiosarcomas are still exposure to VCM and other industrial materials, iatrogenic exposure to colloidal thorium dioxide (Thorotrast), androgenic steroid use, chronic arsenic ingestion, and exposure to radium. Thorotrast was used as a radiocontrast material, but its use was stopped shortly after following several reports of organ damage and fatalities [44,45]. Studies have also shown an association between cancer and exposure to diethylstilbestrol, urethane, cyclophosphamide, and oral contraceptives, which may be considered confounding factors. Due to environmental exposure, hepatic angiosarcomas have a prolonged latency period of 10–40 years, although it seems that the latency period can be estimated to be about 20 years for PVC in most of the cases.

## 5. Conclusions

Far from being catastrophic, we still need to be realistic and advocate for children. There is no safe harbor if we do not take the environmental impact of chemicals seriously, considering that something may be looming over the territories of East Palestine, draining water from the soil and the commercialized livestock. Health officials should monitor the area closely. There is an enlarged, distorted, and indistinct form of opprobrious fate for polluted areas worldwide, and incidents may not be reported as they should in all countries. A close follow-up by public health officials should be considered in Ohio and the surrounding states where water is drained from these geographic areas.

## Data Availability

Publicly available datasets were analyzed in this study at the ToxNet website. Readers are welcome to contact the author for further information if necessary.
